# Monthly Summary

**Published:** 1873-11

**Authors:** 


### MONTHLY SUMMARY.

---

*A Rare and Extraordinary Case.*—The *Irish Hospital Gazette* records an extraordinary case recently brought before the Dublin Pathological Faculty by Professor R. W. Smith of Dublin University. The disease under which the woman succumbed, whose skeleton he exhibited, was one of rare occurrence, and difficult alike to diagnose, treat, or even name. At the time of her death the woman was 45 years old. Fifteen years previously she had been sent to jail for some offense, which was probaly committed while insane, as shortly afterward she was transferred to a lunatic asylum. During the first ten years of her residence there, nothing remarkable about her was noticed, and she was employed in washing the floors, etc. At the end of this period she ceased to be able to work, and remained in bed for the remaining five years of her life, gradually becoming more feeble, and dwindling away in statue until she became about one half of the height she was originally. She did not complain of any pain; her limbs became coiled up in every possible shape, and she seemed gradually to disappear from off the face of the earth. She died possibly from constitutional disease of the osseous system. He [Professor Smith] however, looked upon the condition of the bones not as a disease, but as a manifestation of an as yet unknown diseased condition. Professor Smith had weighed all the bones individually; the total weight of the skeleton [including the cranium] was two and one-half pounds, which equaled about fourth part of the weight of a child at birth. The bones were extremely light, soft, fragile, and atrophied in every respect. The number of fractures were prodigious. The ribs were in a hundred fragments. The head of the humerus was bent; the fibulæ were curved; the thigh bones and pelvis were huddled up together; and the bones of the vertebræ thinned and worn away across the front of their bodies. The lower jaw was atrophied and broken into three fragments; the base of the skull was cribiform all through; and he [Professor Smith] believed that if the woman had lived longer not a vestige of a bone in her body would have been left. As to the nature of this disease he [Professor Smith] believed that it was identical with rickets occuring in the adult, and although that opinion might appear heretical to some, yet he was glad to find that in the last volume of Trosseau's Lectures on Clinical Medicine that that distinguished author had expressed his opinion that osteomalacia and rickets were one and the same disease.

---

*Treatment of Salivation by Atropia.*—Heidenheim has recently investigated the action of atropine on the salivary secretion, and has shown its effects to be [1] to annul the influence of the corda tympani upon the secretion, but [2] not to interfere with its influence upon the circulation, nor [3] to prejudice the secretion by irritation of the sympathetic, thus not destroying the gland-cells. Following H's investigations, Ebstein has applied atropine to the treatment of a case of salivation with remarkable success.

A patient was admitted to the hospital at Breslau, who some months previously had suffered an attack of hemiplegia. At the time of admission traces of the paralysis still remained, with some drooping and partial immobility of left half of the mouth. From the corner of the mouth saliva flowed profusely, and was too copious to be accounted for merely by the paralysis of the orbic. oris. According to the patient's statement, this discharge had persisted for about a month, coming on some months after the occurence of the hemiplegia. Some drooping had accompanied the paralytic stroke at first, but afterwards this ceased.

The atropine was administered at first in pills, each containing 0.0005 atrop. sulph. From one a day the dose was gradually increased to 4 pills [0.002 pro. die.] The discharge of saliva was at the beginning 300 c. c. daily. With the increase of the dose the discharge gradually diminished to 275, 100, and 90 c. c. though it did not cease entirely. On stopping the drug the saliva flowed as at first. The atropia was next administered subcutaneously over the submaxillary gland of the paralyzed side; 0.0003 gramme injected had no effect. The dose was increased to 0.0006, and by degrees to 0.0016. One or two minutes after injecting 0.0006 the patient noticed that his mouth was drier, and in from 5 to 7 minutes the flow had ceased entirely. After the dose of 0.0016 there was no saliva from 4 o'clock P. M. till 6 the next morning. Afterwards the flow recurred. When the injection was made over other parts of the body the effect was delayed to about twice the length of time. This was also the case when the atropine was dropped into the conjunctival sac.

The writer believes that the proper narcotic for salivation is atropia, and in conclusion thinks he can "recommend" its use in the treatment of salivation to his colleagues with good conscience."—*Wilh. Ebstein, Berl. Klin. Wochenschr.*

---

*Hypertrophy of the Tongue.*—Dr. G. Mass reports that five cases of hypertrophy of the tongue have occurred during the past year in the surgical clinique at Breslau. The enlargement was in each case congenital, sometimes involving the entire organ, at others being limited to a latteral half. The part affected was in each case removed by means of the galvano-caustic ligature. The microscopical examination of the removed part showed that in one instance (that of a child two months old,) the enlargement was the result of simple hyperplasia of all the textures of the tongue. In three other cases there was found to be a new formation connective tissue and blood-vessels, so that the tongue was enveloped in a spongy cavernous mass. This formation of new texture had attained the greatest magnitude in the case of a patient twenty-one years old, and was the least marked in the case of a child three years old. The writer concludes that hypertrophy of the tongue begins always with simple hyperplasia, to which is afterwards added, as a secondary lesion, an increased development of the connective tissue and blood-vessels—this abnormal growth being stimulated by the pressure the enlarged organ receives from the surrounding parts, the pressure being so great in some instances as to force it from the mouth.

[*Note.*—Hypertrophy was the term formerly employed to indicate any abnormal enlargement of the body. The distinction indicated by the term *hyperplasia* was first made by Virchow to represent an abnormal enlargement arising from an increase in the number of the original elements. "Hypertrophy" was then limited to that form of enlargement depending upon the increase in the size of the primary elements, such for instance, as is seen in the pregnant uterus.]—*Boston Med. & Surg. Journal.*

---

*Poisonous Character of Nitrous Oxide.*—In the *Archives de Physiologie* Drs. Joylet and Blanche publish the results from their experiments on this subject:—

Chemically pure nitrous oxide will not support the respiration of either animals or plants, as they cannot decompose the gas. When breathed in a pure state by animals, it causes asphyxia and death, with all the symptoms usually occasioned either by strangulation or by the respiration of an inert gas, such as nitrogen or hydrogen. Nitrous oxide causes death in nearly the same time as these other asphyxiating agents. Nitrous oxide has no special anæsthetic action. The anæsthesia which it may produce when inhaled in a pure condition is only due to want of oxygen in the blood. Insensibility appears when the oxygen in arterial blood is reduced to less than 2 or 3 per cent. Arterial blood is then very dark, and contains 30 to 40 per cent of nitrous oxide. Animals can live and show no alteration of sensibility while breathing mixtures of nitrous oxide and oxygen, in the same proportion as nitrogen and oxygen in air. The arterial blood then contains about 30 to 35 per cent. of nitrous oxide. Birds placed under a bell-jar filled with this mixture behave exactly like those placed in a jar of the same size filled with air, and die after having exhausted the oxygen, to a similar extent and formed a similar amount of carbonic acid. As nitrous oxide is an irrespirable gas, and does not possess the anæsthetic properties which have been attributed to it, the authors conclude that its employment cannot but be dangerous, and ought, on this account, to be excluded from medical practice.—*Med. & Surg. Reporter.*

---

*Ice in the Treatment of Facial Neuralgia.*—M. Wenteritz reports a case of very intense facial neuralgia which resisted every mode of treatment, that finally yielded to the application of ice along the affected side of the face laid on at periods of five minutes. The pain disappeared in twelve hours and after an interval now of ten months, has not returned.—*Med. Press.*

*Bleaching Wax*—Wax is freed from its impurities and bleached by melting it with hot water or steam in a tinned copper or wooden vessel, letting it settle, running off clear supernatant oily-looking liquid into an oblong trough with a line of holes in its bottom, so as to distribute it upon horizontal wooden cylinders, made to revolve half immersed in cold water, and then exposing the thin ribbons or films thus obtained to the blanching action of air, light and moisture. For this purpose the ribbons are laid upon long webs of canvas stretched horizontally between standards, two feet above the surface of a sheltered field, having a free exposure to the sunbeams. Here they are frequently turned over and covered by nets, to prevent their being blown away by winds and watered from time to time. Whenever the color of the wax seems stationary, it is collected, remelted, and thrown again into ribbons upon the wet cylinder, in order to present new surfaces to the blanching operation. If the weather proves favorable, the wax eventually loses its yellow tint. Neither chlorine, nor even the chlorides of lime and alkalies, can be employed with advantage to bleach wax, because they render it brittle and impair its burning qualities.—*Am. Artisan.*

---

*An Ointment for Neuralgia.*—Dr. J. Knox Hodge recommends the following as an application which will relieve facial or any other neuralgia almost instantaneously. He advises his friends "to send around to the druggists and try a bottle."

R.—Albumen of egg, oz. j. Rhigolene, dr. iv. Oil of peppermint, dr. ij Collodion, Chloroform, aa dr. j.—M.

Agitate occasionally for 24 hours, and by gelatinization a beautiful semi-solidified, opodeldoc-looking compound results, which will retain its consistency and hold the ingredients intimately blended for months.

In the above we find a local agent of signal potency in all neuralgic affections, and for the relief of pain generally.

Apply by smart friction with the hand, or gently with a soft brush mop along the course of the nerve involved.—*Georgia Companion.*—*The Clinic.*

---

*Remarks on Methylene.*—Dr. Richardson, speaking of this anæsthetic as rapid in its effect, says, that "from one to two drachms will induce, in the space of a minute and a half to two minutes, sufficient insensibility for a short operation, while from two to six drachms are sufficient for the production of prolonged anæsthetic sleep." The sleep induced is very gentle, and rarely attended with convulsive movement; vomiting is less frequent than from chloroform, ether, or bichloride of methylene.

*Treatment of Burns.*—Dr. Buck's burn-mixture, in use at Bellevue Hospital, N. Y., [*Med. Record,*] is made by dissolving two ounces gum tragacanth and four ounces gum Arabic in a pint of water, and adding a pint of molasses. Spread on with a brush, and repeat when needed. Common white paint is also used, spread over the burnt surface. Lycopodium is applied by some surgeons over the mucilage.

Pneumonia is sometimes treated with sulph. quiniæ gr. v. three times a day, and good results are reported. But pnemonia is a wonderful plaything for statistics.—*Pacific Med. and Surg. Journal.*

